# Involving hard-to-reach ethnic minorities in low-budget health research: lessons from a health survey among Moluccans in the Netherlands

**DOI:** 10.1186/s13104-016-2124-1

**Published:** 2016-06-21

**Authors:** Adee J. Bodewes, Anton E. Kunst

**Affiliations:** Department of Public Health, Academic Medical Centre, PO Box 22660, 1100 DD Amsterdam, The Netherlands

**Keywords:** Moluccans, Health surveys, Recruitment, Response rates, Ethnic minorities

## Abstract

**Background:**

There is little evidence on which strategies are effective in recruiting minority groups in low-budget health surveys. We evaluated different recruitment strategies for their impact on response rates in a hard-to-reach minority population in the Netherlands.

**Methods:**

We conducted a health survey in 19 Moluccan districts (MDs). Each MD had its own set of recruitment strategies, such as information meetings, involving social or local media, involving community organizations, and door-to-door collection. The association between recruitment strategies and MD-specific response rates was assessed with logistic regression analysis.

**Results:**

The overall response rate was 24 %, and varied from 9 to 58 %. Higher rates were obtained when the strategy included door-to-door collection (OR 1.57) and ‘active’ key informants (OR 1.68). No positive associations with response rates were observed of the other strategies.

**Conclusions:**

The overall low response rate in this study may be due to high levels of distrust, segmentation within the community and high respect for privacy among Moluccans. Our study shows that in such communities, response may be increased by a highly personal recruitment approach and a strong commitment and participation of community key-figures.

## Background

Recruiting and involving minority participants in health surveys present important challenges [1, 2]. Recruiting participants is a time and resource consuming part of survey research among minority groups [3]. Various studies have experienced difficulties in penetrating minority communities, resulting in low participation rates and poor representativeness of the respondents for the target population [4–9]. Studies among ethnic minorities therefore need to improve the recruitment methods and take into account characteristics of the target population in order to achieve satisfactory response rates [4].

Many recommendations have been made regarding strategies to involve and recruit minority participants in research [4, 10–12]. Communication with the people and building relationships is stated to be foundational in research among minority communities. Recommended strategies include building trust, increasing visibility of the investigators, involving community advisors, sharing insights with the target group, and forming social networks within the community [13–17]. Recruiting minority participants can be done in various ways, including door-to-door enumeration, community approaches such as community events, and snowball methods [3, 18–21]. Avoiding distrust and creating trust is a common theme in different strategies to recruit minority participants in research [3, 16, 22–24].

One general approach has not been identified for maximizing response rates among minority participants. Response rates may strongly vary among studies that employ similar recruitment strategies though among different minorities. For example, the studies of Khan et al. and of Tucker et al. both used snowball sampling but achieved response rates of respectively 48.3 and 7.2 % [18, 20, 25–27]. Recruitment strategies such as face-to-face recruitment, telephone follow-up and the use of incentives appear to increase the response rate among several minority groups [25, 28, 29].

The effectiveness of different strategies in reaching and involving minority groups is particularly important in low-budget health surveys. In such surveys, strategies that require a huge input in terms of money or manpower may not be feasible. In low-budget studies, therefore, a critical question is which specific recruitment strategies require few resources but may nonetheless be effective in enhancing response rates. This study aims to provide systematic evidence to support such choices. Unfortunately, there is yet limited available empirical evidence to determine which low-budget strategies could be most effectively applied among minority groups.

In the Netherlands, most of the research on health of minorities focuses on the largest minority groups of Moroccans, Turkish and Surinamese. One hard-to-reach minority group has been largely ignored until now: the Moluccans. In 1951, a group of approx. 3000 Moluccan soldiers with their families, who served in the Royal Dutch East-Indies Army during the WOII, were transferred to the Netherlands [30]. Soon after arrival, those who were unable to return to the new Republic of Indonesia were turned into exiles. Today, the total number of Moluccans is estimated to be about 50,000 [30], of which approx. one third is 3rd generation. Still today, Moluccans have some degree of social disadvantage; generally Moluccans are lower educated and have lower-ranking occupations than the Dutch autochthonous population [31]. By the start of this study, there was very limited evidence regarding the health status of the Moluccan population. Recent studies demonstrate that Moluccans have high prevalence rates of hypertension and ischemic heart disease as compared to the native Dutch [30, 32]. Given their lower socioeconomic position and persistent problems of integration [33] other types of health problems might as well be more prevalent among Moluccan residents.

A particular feature of this minority group is their residence in so-called ‘Moluccan districts’ (MDs). These districts are located at the outskirt of more than 60 Dutch villages and towns distributed throughout the Netherlands and they accommodate the majority of the first and second generation Moluccans [34].

The Moluccan community may be particularly difficult to access. The Moluccan culture is one with a strong adherence to traditional values, social hierarchy, family bonds and Malay language [30]. Respect for privacy is important, and personal affairs are not easily disclosed to those outside the family. Furthermore, political history has left its scars within the community, resulting in distrust towards the Dutch government and also towards non-governmental organizations [33]. Finally, the Moluccans wait-and-see attitude restricts their close involvement in research [33]. As a consequence, recruiting Moluccans may be challenging. Unfortunately, to our knowledge, experiences in recruiting Moluccans in health surveys, if any, are not documented.

The general aim of this paper is to report on the recruitment strategies that we applied with the aim to increase the response rate to a health survey among Moluccans in the Netherlands. This survey was carried out in 19 MDs. In the different MDs, we applied zero to five recruitment strategies, which were administered in different combinations. The specific aim of this study was to evaluate these different combinations of strategies with regard to their effect on response rates.

## Methods

### The survey

The survey, conducted from August 2012 to March 2013, aimed to obtain national-level estimates of the health situation of Moluccans, aged between 30 and 65 years, living in Moluccan districts (MDs). We selected 19 of the 60 existing MDs throughout the Netherlands. The selection was based on three criteria: the population size of each MD should be relatively large (more than 50 eligible persons), key informants could be approached easily, and all geographic regions and religious groups (mostly Christian, few Muslim) should be represented.

Lists of addresses were collected via key informants. They created a list of potential respondents and their addresses, taking into account three guidelines: respondents had to be of Moluccan origin, respondents had to live within or close to the MD. We also included persons with only one parent with Moluccan roots. Selected persons received a health questionnaire delivered at their address by hand or post.

The health questionnaire was based on the *POLS* (Continuous Survey of Living Conditions) questionnaire of Statistics Netherlands [35]. Socio-demographics, self-reported health, healthcare utilization and health-related behaviours were the core topics of the questionnaire. The questionnaire contained approximately 80 questions and could be completed within 20 to 25 min. The questionnaire was translated into the Malay language for those who would not master the Dutch written language. Questionnaires could be filled in during a face-to-face interview, using an Internet questionnaire, or using the paper-and-pencil method. All non-respondents received a reminder after 2 weeks.

In each MD, key informants of Moluccan roots were closely involved in the survey fieldwork. Most of them were members of the local Moluccan neighbourhood council or of the board of the Moluccan community centre. In consultation with each key informant, we developed a set of recruitment strategies that could be implemented with the time and resources available within their own MD.

### Recruitment strategies

The available recruitment strategies can be categorized into “direct” and “indirect”. A direct strategy involved personal contact with the potential participants with the aim to provide them with personalized information regarding the survey. In an indirect strategy, we did not approach people individually but we provided information about the survey to the community as a whole, including information on how to contact the researchers for further information.

Direct recruitment strategies included information meetings and door-to-door collection of questionnaires. Information meetings lasted approximately 3 h and aimed to inform the participating community members about the survey and to discuss the survey design, possibilities for follow-up research, and requirements to dissemination. In door-to-door collection, key informants would go door-to-door in the community to collect the questionnaires.

Indirect recruitment strategies included use of announcement letters, social media, local media, and community organizations. Announcement letters were sent by post or by email to each Moluccan person listed in the selected MD. This letter explained the purpose of the research, introduced the involved community key informants and presented the researchers. Social media included the website of local community centre and the local Facebook page. Local media included local newspapers and the local radio station. At both media, we placed short messages to inform Moluccans about the research and to remind the invited community members to participate. Finally, we could involve the Moluccan church in two MDs and the local general practitioners (GP) centre in one MD. Their involvement consisted of providing a recommendation letter in support of the study. Contact with these community organizations were initiated by the researchers or by the key informants.

To stimulate response we introduced a financial reward of three travelling vouchers with a value of €500,- and twenty dinner vouchers of €20,-. After the fieldwork was closed, the winning persons were randomly selected from all respondents.

### Analysis

For each MD we collected data on the number of key informants, the number and type of recruitment strategies that were implemented, the number of questionnaires that were distributed, and the number of questionnaires that were returned to the researchers. The response rate for each MD was calculated by dividing the number of returned to the number of distributed questionnaires. With regard to key informants, we also measured their level of activity during the recruitment process. Key informants were considered to have been ‘active’ if they made major effort during the recruitment process, e.g., by recruiting respondents via door-to-door enumeration. A key informant was considered ‘inactive’ as he/she only provided the address list and/or distributed reminders in the community.

In a first step, univariate analysis was performed to calculate the response rate according to the presence of each recruitment strategy. For each strategy, we distinguished between “exposed” and “non-exposed” MDs and we calculated the total number of questionnaires that were distributed and returned in the exposed and non-exposed group, respectively. A similar calculation was performed to calculate response rates in relationship to the presence of ‘active’ key informants.

In further analyses, we assessed the independent association of each strategy to the response rates by using multilevel and multivariate logistic regression analysis, with individual invited participants at the lower level and MDs representing the higher level. The dependent variable was whether or not an invited participant had responded. Independent variables were dichotomous variables measuring the presence of specific recruitment strategies at the level of MDs. We also include the number of key informants as continuous variable, and the presence of ‘active’ key informants as dichotomous variable. Odds ratios (ORs), the corresponding 95 % confidence intervals (95 % CI) and p values were derived from the regression estimates. A p value ≤ 0.05 was considered to indicate statistical significance. Analyses were performed using the statistical program R i386 3.0.1.

## Results

The number and combination of recruitment strategies used within each MD are shown in Table 1. Overall, the number of implemented recruitment strategies varied from one to four. Exceptions were Middelburg (where no recruitment strategy was applied) and Vaassen (where five recruitment strategies were applied). Only three pairs of MDs applied the same combination of recruitment strategies: Nistelrode and Oost-Souburg/Vlissingen; Maastricht and Waalwijk; and Vught and Ridderkerk. The number of key informants involved varied between MDs. In Capelle aan den IJssel, no key informants were involved, and we had to organize the recruitment ourselves. Also the activity level of the key informants varied between the MDs (see Table 1).

**Table 1:** Overview of the implemented recruitment strategies in each Moluccan district

Moluccan district	Strategy (N)	Key informants		Indirect recruitment strategies				Direct recruitment strategies	
		N	Active^a^	Announcement letter	Local media	Social media	Community organizations	Information meeting	Door-to-door
Maastricht	2	5	Yes	X					X
Den Helder	2	1	Yes	X				X	
Bovensmilde	4	4	Yes	X			X	X	X
Lunteren	3	3	Yes	X	X	X			
Breukelen	3	1	No	X	X			X	
Waalwijk	2	1	Yes	X					X
Nistelrode	4	2	Yes	X	X	X		X	
Middelburg	0	1	Yes						
Oost-Souburg/Vlissingen	4	1	No	X	X	X		X	
Zwolle	2	3	No	X					X
Vught	1	1	Yes			X			
Assen	2	5	No	X		X			
Capelle a/d Ijssel	4	0	No	X	X	X			X
Ridderkerk	1	2	No			X			
Groningen	1	2	No	X					
Hoogeveen	2	2	No			X	X		
Vaassen	5	2	Yes	X	X	X	X	X	
Wierden	2	1	No			X		X	
Breda	1	1	No	X					

^a^Activity level: key informants were considered to be ‘active’ if they made major effort during the recruitment process (see text for the details)

Table 2 presents an overview of the response rates achieved in each MD. The total response rate was 24 %. The response rates of individual MDs varied from 9 to 58 %. The MD of Maastricht had the highest response rate (58 %), followed by Den Helder (46 %) and Bovensmilde (38 %) (see Table 2).

**Table 2:** Overview of the distribution of respondents, questionnaires and response rates for each Moluccan District

Moluccan district	Respondents (N)	Questionnaires distributed (N)	Response rate (%)
Maastricht	80	137	58
Den Helder	35	76	46
Bovensmilde	63	165	38
Lunteren	68	203	34
Breukelen	21	67	31
Waalwijk	25	80	31
Nistelrode	46	179	26
Middelburg	67	269	25
Oost-Souburg/Vlissingen	32	141	23
Zwolle	43	198	22
Vught	26	126	21
Assen	45	219	21
Capelle aan den Ijssel	44	220	20
Ridderkerk	13	66	20
Groningen	18	96	19
Hoogeveen	20	115	17
Vaassen	29	232	13
Wierden	18	151	12
Breda	18	212	9
*On request* ^a^	*4*	*4*	–
Total	715	2956	24

^a^On request revers to respondents who were not recruited via the presented recruitment strategies but via verbal transmission

For each recruitment strategy, Table 3 presents the number of exposed and non-exposed MDs and the corresponding response rates. Higher response rates were obtained in MDs where we applied door-to-door collection of questionnaires (31.8 vs. 21.2 %) and where we sent an announcement letter (25.5 vs. 19.8 %) compared to MDs where we did not apply these strategies. No higher response rates were observed in MDs where we utilized local and social media, where we could involve community organizations, or where we organized an information meeting. Response rates were higher in MDs where key informants were actively involved in the recruitment process (30.0 vs. 18.1 %) (see Table 3).

**Table 3:** Association between recruitment strategy and response rate: comparing exposed and non-exposed districts, to different strategies

	Number of districts (N)		Response rate (%)	
	Exposed	Non-exposed	Exposed	Non-exposed
Indirect recruitment strategy				
Announcement letter	14	5	*25.5*	19.8
Information meeting	7	12	24.1	24.1
Local media	6	13	23.0	24.6
Social media	10	9	20.6	28.5
Direct recruitment strategy				
Door-to-door collection	5	14	*31.8*	21.2
Involvement of community organizations	3	16	21.8	24.5
Other strategies				
“Active” key informants^a^	9	10	*30.0*	18.1

Response rates with more than 5 % increase between exposed and non-exposed communities are presented in italics

^a^Key informants were considered to be ‘active’ if they made major effort during the recruitment process (see text for the details)

Table 4 presents the results of the regression analysis in terms of odds ratios (ORs) with their corresponding 95 % confidence intervals (95 % CI). Response rates were positively associated with door-to-door collection (OR 1.57; 95 % CI 1.01–2.43), but inversely associated with the involvement of community organizations (OR 0.53; 95 % CI 0.32–0.88). The number of key informants is positively related with response rates: involving one more key informant in the recruitment process increases the OR with 0.25 (95 % CI 1.07–1.45). The involvement of key informants who were ‘active’ is also associated with higher response rates (OR 1.68; 95 % CI 1.18–2.38). Excluding the variable on door-to-door collection from the model did not substantially change the ORs corresponding to the other recruitment strategies. No independent associations were found for variables representing, respectively, the use of announcement letters, information meetings, local and social media (see Table 4).

**Table 4:** Association between recruitment strategy and response rate: results from multivariate and multilevel logistic regression

	OR (95 % CI)			
	Model 1	Model 2	Model 3	Model 4
No strategy (ref.)	1.00	1.00	1.00	1.00
Announcement letter	1.03 (0.47–2.25)	0.56 (0.26–1.17)	0.69 (0.36–1.29)	0.83 (0.42–1.62)
Information meeting	1.27 (0.69–2.35)	1.41 (0.86–2.31)	1.31 (0.86–1.98)	1.16 (0.74–1.80)
Local Media	1.08 (0.49–2.39)	1.89 (0.91–3.94)*	1.52 (0.81–2.83)	1.46 (0.73–2.93)
Social Media	0.71 (0.36–1.40)	*0.52 (0.29–0.93)*	0.64 (0.38–1.05)	0.58 (0.33–1.01)*
Door-to-door collection	1.69 (0.87–3.25)	1.64 (0.97–2.79)*	*1.57 (1.01–2.43)*	–
Involvement of community organizations	0.75 (0.37-1.54)	0.58 (0.31-1.06)*	*0.53 (0.32–0.88)*	0.60 (0.34-1.05)*
Key informant (cont.)^a^	–	*1.32 (1.11–1.58)*	*1.25 (1.07–1.45)*	*1.25 (1.06–1.48)*
Active key informant^b^	–	–	*1.68 (1.18–2.38)*	*1.72 (1.16–2.53)*

Model 1: single recruitment strategies

Model 2: model 1 + key informants

Model 3: model 2 + active key informants

Model 4: model 3—door-to-door collection

A p value ≤ 0.05 is presented in italics

* A p value ≤ 0.1

^a^The continuous variable of key informants represents the increase in OR corresponding to one additional key informant contributing to the recruitment process

^b^Key informants were considered to be ‘active’ if they made major effort during the recruitment process (see text for the details)

## Discussion

The aim of this paper was to assess the effectiveness of several recruitment strategies to increase the response rate in a low-budget health survey among a hard-to-reach minority community in the Netherlands. We achieved a total response rate of 24 %, which varied from 9 to 58 % between the Moluccan districts (MDs). Response rates were positively associated with door-to-door collection of questionnaires, and with the involvement of ‘active’ key informants in the recruitment process.

The overall response rate of 24 % was disappointingly low [36]. This response rate is lower than the reported response rates achieved in studies among other minority groups in the Netherlands and which are generally above 40 % [37–40]. Between the MDs, response rates were achieved ranging from 9 to 58 %. The demographic composition varied between the MDs. Closer inspection showed this composition is not clearly correlated with overall response rates, nor with the application of specific recruitment strategies. Possible explanations for this low response can be found in the study design and in characteristics of the Moluccan community.

With regard to study design, the limited time and financial resources available to this study could have negatively influenced the response rate. No prior experiences were available in recruiting respondents in population surveys in these MDs. The total fieldwork period was limited to about 8 months, during which the address lists had to be created and recruitment strategies had to be developed and implemented in 19 MDs throughout the Netherlands. The recruitment period per MD varied from 3 to 7 months. Due to limited financial resources, we could not apply resource-demanding strategies that are potentially effective, such as telephone follow-up and substantial financial rewards to all respondents. Finally, for reasons related to their unique history [33], Moluccans cannot be identified as such in the Dutch population registry, and we had to invest considerable time in creating address lists that included all eligible persons.

As to the role of the Moluccan community itself, several barriers became apparent during the survey. Our conversations with community members revealed high levels of distrust towards this study despite the key researcher (AB) being of Moluccan descent. This distrust reinforced pre-existing reluctance among Moluccans to disclose personal information to other people. Moreover, the subject ‘health’ appeared to be a taboo topic within the Moluccan community, rather than a subject that fostered interest and participation. A case of disease is kept within the family and is not spoken of within the community. At a wider community level, we observed that response was affected by a high degree of segmentation within the local communities. A strong sense of individualism and orientation of single families affected the residents’ willingness to contribute to community-oriented activities. This also affected the survey: while we emphasized the value of the survey as a ‘public’ good, potential respondents tended to be looking for the personal benefits that they could gain from participation.

Despite these hurdles, we observed some recruitment strategies to be positively associated with response rates. Door-to-door collection of questionnaires seemed an effective strategy in raising response among Moluccans. Other than in the general Dutch population [41] and most of its minority groups, simply sending a questionnaire was insufficient in achieving a high response rate among most Moluccan residents. A possible reason is that within the Moluccan community, personal contact is essential, be it via telephone, personal visits, or meeting at special occasions. We experienced that non-personal communication routes such as post, e-mail, Facebook and Twitter were not common ways to contact Moluccans, especially those of the first generation (about 65 years and older) and second generation (between 44 and 64 years). Door-to-door collection may be essential to achieve the personal contact that is needed to create trust and mutual understanding [3, 14, 42].

Involving key informants in the research process was associated with increased response rates [43, 44]. Most of our key informants represent the needs and wishes of the community members and were considered to be the backbone of the local community [3]. Most studies place key informants in an ‘advisory’ role within the research process, whose role is to inform the researchers about the community [11, 16, 45]. In our case, however, key informants played an active role in recruiting respondents, e.g., by door-to-door enumeration or organizing community meetings in which the questionnaire could be filled in jointly. In most other studies, door-to-door enumeration is done by researchers themselves or by recruited minority interviewees [44, 45]. In our case, the presence of key informants may have increased the impact of house visits on individual respondents.

Response rates were lower in MDs where we involved community organizations, more specifically churches. This result has to be interpreted with caution because churches were involved in only three MDs. We expected a positive impact as the majority of the Moluccan community is strongly religious [14]. The lack of evidence for such a positive impact may suggest that the involvement of churches had limited added value. This finding is in contrast to other studies [46, 47], and might be related to segmented nature of Moluccans communities, high levels of distrust, and wish for privacy among Moluccans. The church board may not be fully trusted and Moluccans may be apprehensive that the information from the surveys may be used for personal gain by the church board.

In Vaassen, the response rate was very low (13 %) even though we applied five recruitment strategies and involved active key informants. This low response might be due to the fact that this is one of the most traumatized Moluccan communities. In 1976 Moluccans in this area were displaced contrary to their will—an event that led to a violent encounter with local authorities [48]. This event has left deep scares within the local community until today.

## Conclusions

To conclude, our experiences underline that increasing response rates among hard-to-reach minorities is challenging in surveys that have few resources for individual communities. Before starting, an analysis of the community structure is needed to identify possible recruitment obstacles, such as distrust and social segmentation. The involvement of active key-informants may be critical in this phase. While developing recruitment strategies focusing on community values and public good [49] may be effective in communities with strong social cohesion, such an approach may be insufficient in segmented, individualised communities. In such cases, recruitment methods may instead need to emphasize the personal benefits of participation and use personalized strategies such as door-to-door recruitment.
